# Naturalistic movie viewing is an effective functional localizer of the fusiform face area in adolescents with and without autism

**DOI:** 10.1162/IMAG.a.1209

**Published:** 2026-04-08

**Authors:** Clara J. Steeby, Gillian N. Miller, Alberto Castro Palacin, Alexander L. Cohen

**Affiliations:** Department of Neurology, Boston Children’s Hospital, Harvard Medical School, Boston, MA, United States; Center for Brain Circuit Therapeutics, Brigham and Women’s Hospital, Harvard Medical School, Boston, MA, United States

**Keywords:** fMRI, autism spectrum disorder, functional localization, naturalistic stimuli, fusiform face area

## Abstract

Task-based “localizer” neuroimaging protocols are often used to identify specific functional areas in individual participants. Conventionally, these tasks can be unengaging, leading to increased motion and scan fatigue, especially for populations which may struggle with lengthy scans. Dynamic, naturalistic stimuli, like entertaining movies, offer a potential alternative with higher participant engagement and improved data quality. Here, we evaluated whether a short Pixar movie, *Partly Cloudy*, could serve as a reasonable and useful replacement for a traditional fusiform face area (FFA) localizer in a group of adolescents with and without autism spectrum disorder (ASD). We found that individualized FFA localizations derived from a Pixar movie, *Partly Cloudy*, were largely consistent with those produced by a traditional localizer. Peak activation locations showed no difference between localizer types; measures of activation pattern and magnitude were also largely the same, except that the occipital face area (OFA) demonstrated more task than movie activation and the parahippocampal place area (PPA) demonstrated more movie than task activation. However, applying these FFA individualized ROIs (iROIs) to independent task data revealed that traditional localizer iROIs still produce more face-selective activations. We also demonstrated that the movie viewing helps to reduce motion levels in both adolescents with and without autism relative to the traditional localizer task. Hence, while they may not localize face-selective regions of the FFA as robustly as traditional task-based localizers, movie localizers may be an appropriate, or even preferable, choice for fMRI studies in populations which struggle to tolerate lengthy scans, such as individuals with autism, and in paradigms that must rapidly localize multiple functional regions.

## 1 Introduction

Studying distinct functional areas in the brain with neuroimaging techniques at an individual level requires a precise approach, as the exact location of specific functional areas can vary across individuals (Saxe et al., 2006). Conventionally, this is achieved with a task known to preferentially elicit activity in the given area, such as a “faces versus houses” task to localize the fusiform face area (FFA). These localizers can be constructed from different types of task conditions, such as passive viewing or n-back memory tests, without impact on the location of activations (Berman et al., 2010). They can also involve dynamic stimuli, such as short clips of faces, which have been shown to be more effective at activating many visual processing regions (Fox et al., 2009; Pitcher, Dilks, et al., 2011). However, traditional task-based localizers can often be dull, require sustained attention, and increase the time burden of the scan session—challenges that are especially salient when working with children or clinical populations, such as individuals with autism spectrum disorder (ASD). Using naturalistic stimuli**,** such as made-for-entertainment movies, may provide an alternative to traditional task-based localizers that are both more engaging and result in lower levels of motion. Naturalistic stimuli are rich, dynamic, and composed of multiple elements with complex time courses that evoke more naturalistic patterns of neural responses compared to traditional fMRI tasks (Vanderwal et al., 2019). The literature suggests that using engaging naturalistic stimuli, for example, movies, may decrease the amount of motion as compared to rest (Frew et al., 2022; Greene et al., 2018; Vanderwal et al., 2015) and compared to simple tasks (Cantlon & Li, 2013).

During movie watching, about 30% of the cortex demonstrates concordant activity across individuals. Specifically, areas of the cortex involved in visual and auditory processes, as well as social cognition areas, show high intersubject correlation during movie watching (Hasson et al., 2004; Keles et al., 2024; Lerner et al., 2021). The overall concordance of cortical activity increases from childhood to adulthood; in adolescence, areas of the ventral temporal cortex, including face processing regions, show highly synchronized activity during movie watching (Lerner et al., 2021). This suggests that studying brain responses during movies is a reliable way to investigate cognitive processes and category-specific areas across individuals. As such, movies have begun to be explored as functional localizers to identify category-selective topographies and networks in individuals. In both adults and children, movies have successfully been used as functional localizers to localize face-, object-, scene- preferring areas (Kamps et al., 2022) as well as theory of mind and pain networks (Jacoby et al., 2016; Richardson et al., 2018). Jiahui et al. (2023) also used connectivity hyperalignment to map face-, body-, scene-, and object-preferring areas in individuals who watched different movies**.**

Movies are an especially useful fMRI “task” when studying populations that struggle with motion during scanning. It has been well-documented that individuals with autism demonstrate increased motion during fMRI scanning paradigms, particularly during task-free rest (Cox et al., 2017; Nebel et al., 2022; Starck et al., 2013; Tyszka et al., 2014). In addition, autistic participants are at greater risk of scan fatigue and the stressful MRI environment can limit the accessibility of participation, contributing to unrepresentative sampling (Sader et al., 2024; Stogiannos et al., 2023). Thus, using naturalistic stimuli as a functional localizer in autism research may be a good choice for several reasons, including to 1) improve motion, 2) make scans more engaging/less stressful, and 3) increase accessibility to individuals with autism that are traditionally not able to participate in fMRI research.

### 1.1 The present study

A short Pixar movie (~5 minutes) was recently explored as a dynamic, naturalistic functional localizer in neurotypical adults and young children (Kamps et al., 2022). They compared functional selectivity of individualized regions of interest (ROIs) defined from the Pixar movie, from a traditional dynamic localizer task, and from an independent cohort ROI using high-quality adult data. The movie approach outperformed independent cohort-based localization in adults, even when only 2.7 minutes of movie data was available. They also found that movie-derived individual ROIs in young children were highly correlated to task-derived individual ROIs in adults, even as young as 3 years old; however, they did not perform a direct within-subject comparison of movie- versus task-derived ROIs in children. The present study extends this work by investigating the same short made-for-entertainment movie, *Partly Cloudy*, as a functional localizer in a developmental, clinical population known to exhibit high levels of in-scanner motion: autistic adolescents. We define individual ROIs in this population and make a comparison to individual ROIs defined from a traditional static localizer task data in the same participants.

Here, we assess the relative benefit of a movie-based versus task-based functional localizer for localization of the Fusiform Face Area (FFA), a well-studied region known to selectively activate to faces (Kanwisher et al., 1997). We compare FFA localization between a task involving photos of human faces and a cartoon movie involving anthropomorphic faces—stimuli with clear differences yet known to similarly activate the FFA. Evidence suggests that the FFA does not show a preference for dynamic stimuli (Bernstein et al., 2018) or differences in activation between cartoon and human faces (Tong et al., 2000). Some evidence also suggests that autistic individuals process cartoon faces in a more “facelike” manner than real human faces—autistic individuals tend not to show a face inversion effect but do show this with cartoon faces (Rosset et al., 2008), indicating that the movie may even be superior.

Finally, we assess the benefit of movie-based localization for motion reduction, a critical goal when scanning clinical and developmental populations. We present FFA localizations from movie watching and a traditional localizer task, compare the peak activation locations and patterns, as well as compare to an FFA ROI derived from the literature, when applied to an analysis of a passive face viewing task in neurotypical and autistic adolescents.

## 2 Methods

### 2.1 Participants

Forty-nine 13- to 18-year-old adolescents participated in this study. One neurotypical participant was excluded due to high levels of motion, resulting in a final cohort of 48 participants. Twenty-four participants had a clinical diagnosis of autism spectrum disorder (ASD), while 24 participants were neurotypical adolescents (NT). Groups differed in age and full-scale IQ (Table 1). Informed consent was provided by all adult participants; all adolescent participants provided assent and their parents/legal guardians provided informed consent to participate. Participants were recruited from Boston Children’s Hospital via the Translational Neuroscience Center’s Research Participant Registry and from the community via flyers. This study was approved by Boston Children’s Hospital IRB.

**Table 1.** Participant information.

	All (n = 48)	ASD (n = 24)	NT (n = 24)	p
Sex (% F)	14F (29%)	5F (21%)	9F (38%)	n.s.
Age (years)	16.1 ± 1.7	15.4 ± 1.7	16.7 ± 1.4	p < 0.05
WASI-II FSIQ	107.8 ± 15.2	103.5 ± 16.6	114.9 ± 11.9	p < 0.001

WASI-II: Wechsler Abbreviated Scale of Intelligence, Second Edition.

### 2.2 fMRI stimuli

Participants completed a single fMRI session involving (in order): T1w-, T2w-structural imaging, resting state, functional localizer task, resting state, functional localizer task, *Pixar* movie short, resting state, functional localizer task, abstract movie, functional localizer task, and resting state. On average, scans lasted 70-minutes. Reversed phase-encoding fieldmaps were obtained after each functional scan. The localizer task used was a traditional faces versus houses block-design (Haxby et al., 1999) visual attention task, which is well documented as eliciting strong FFA activity; each run was 3.9-minutes in length. The task presentation consisted of four 36-second blocks (upright faces, upright houses, inverted faces, inverted houses) alternating with 18-second rest blocks. Additionally, each stimulus block included one 3-second presentation of a flower. Each task run also began and ended with a rest block, for a total of 5 rest blocks. To ensure alertness during the task, participants were instructed to view the stimuli and respond with a button press when a flower, randomly embedded in the sequence of faces and houses, appeared. Stimuli were black and white racially diverse isolated neutral faces (RADIATE; Conley et al., 2018) and black and white houses, selected to be highly “face-like” (DalHouses; Filliter et al., 2016). Stimuli were shown in upright and inverted blocks. The first movie was *Partly Cloudy*, a 5.4-minute animated Pixar short, played with audio and without the end credits sequence. A small number of participants (n = 3) did not watch the movie with audio, due to head size constraints when wearing headphones in the scanner. A plot description can be found online (https://www.pixar.com/partly-cloudy#partly-cloudy-1). In addition, participants completed four 5.0-minute long resting-state scans while viewing a white fixation cross on a black background and an additional 7.0-minute abstract movie (*Inscapes,* Vanderwal et al., 2015); these data are not analyzed here.

### 2.3 fMRI data acquisition

Whole-brain structural and functional MRI data were acquired on a 3T Siemens MAGNETOM Prisma scanner equipped with a 128-channel head coil located at Boston Children’s Hospital. Functional blood oxygen-level dependent (BOLD) images were acquired with a gradient-echo EPI sequence (TR = 1060 ms; TE = 30 ms; slices = 60, flip angle = 52; EPI factor = 90) with 2.4 × 2.4 × 2.4 mm voxel resolution. T1-weighted structural images were collected in 160 sagittal slices with 1.00 mm isotropic voxels (TR = 1410 ms, TE = 2.06 ms).

### 2.4 fMRI data preprocessing

Results included in this manuscript come from preprocessing performed using fMRIPrep 23.1.3 using standard settings (Esteban et al., 2019, 2025; RRID:SCR_016216) which is based on Nipype 1.8.6 (Gorgolewski et al., 2011, 2017; RRID:SCR_002502).

#### 2.4.1 Preprocessing of B0 inhomogeneity mappings

Fieldmaps were acquired for most participants (n = 44), except in a subset of participants, for whom fieldmaps were not usable due to incorrect acquisition (n = 4). For participants with fieldmaps, a B0-nonuniformity map (or fieldmap) was estimated based on two (or more) echo-planar imaging (EPI) references with “topup” (Andersson et al., 2003; FSL 6.0.7). For participants without fieldmaps, a deformation field to correct for susceptibility distortions was estimated based on fMRIPrep’s “fieldmap-less” approach. A deformation field was created from co-registering the participant’s EPI reference image to the same-subject T1w-reference with its intensity inverted (Huntenburg, 2014; Wang et al., 2017). Registration was performed with antsRegistration (Avants et al., 2008; RRID:SCR_004757), and the process regularized by constraining deformation to be nonzero only along the phase-encoding direction, and modulated with an average fieldmap template (Treiber et al., 2016). This synthetic fieldmap was then used to unwarp the participant’s fMRI data.

#### 2.4.2 Anatomical data preprocessing

Each participant’s T1-weighted (T1w) image was corrected for intensity non-uniformity (INU) with N4BiasFieldCorrection (Tustison et al., 2010), distributed with ANTs (Avants et al., 2008, RRID:SCR_004757), and used as T1w-reference throughout the workflow. The T1w-reference was then skull-stripped with a Nipype implementation of the antsBrainExtraction.sh workflow (from ANTs), using OASIS30ANTs as the target template. Volume-based spatial normalization to one standard space (MNI152NLin2009cAsym) was performed through nonlinear registration with antsRegistration, using brain-extracted versions of both T1w reference and the target template. The following template was selected for spatial normalization and accessed with TemplateFlow (23.0.0; Ciric et al., 2022): ICBM 152 Nonlinear Asymmetrical template version 2009c (Fonov et al., 2009; RRID:SCR_008796; TemplateFlow ID: MNI152NLin2009cAsym].)

#### 2.4.3 Functional data preprocessing

The following preprocessing was performed for each BOLD run using fMRIPrep. First, a reference volume and its skull-stripped version were generated using a custom methodology of fMRIPrep. Head-motion parameters with respect to the BOLD reference (transformation matrices including six corresponding rotation and translation parameters) were estimated before spatiotemporal filtering using mcflirt (FSL; Jenkinson et al., 2002). The estimated fieldmap was then aligned with rigid-registration to the target EPI (echo-planar imaging) reference run. The field coefficients were mapped on to the reference EPI using the transform. BOLD runs were slice-time corrected using 3dTshift from AFNI (Cox & Hyde, 1997; RRID:SCR_005927). The BOLD reference was then co-registered to the T1w reference using bbregister (FreeSurfer) which implements boundary-based registration (Greve & Fischl, 2009). Co-registration was configured with six degrees of freedom. Several confounding time-series were calculated based on the preprocessed BOLD: framewise displacement (FD), DVARS, and three region-wise global signals. FD was computed using two formulations following Power (absoluate sum of relative motions, Power et al., 2014) and Jenkinson (relative root mean square displacement between affines, Jenkinson et al., 2002). FD and DVARS are calculated for each functional run, both using their implementations in Nipype (following the definitions by Power et al., 2014). The BOLD time-series were resampled into standard space, generating a preprocessed BOLD run in MNI152NLin2009cAsym space. All resamplings were performed as a single interpolation step by composing all the pertinent transformations (i.e., head-motion transform matrices, susceptibility distortion correction when available, and co-registrations to anatomical and output spaces). Gridded (volumetric) resamplings were performed using antsApplyTransforms (ANTs), configured with Lanczos interpolation to minimize the smoothing effects of other kernels (Lanczos, 1964).

### 2.5 Motion comparison

We expected that, in comparison to a traditional localizer task, participants would move less during movie viewing. To test this, we compared mean framewise displacement, which is closely related to motion artifacts and is a good estimate of problematic motion (Ciric et al., 2017; Power et al., 2012; Satterthwaite et al., 2012; Van Dijk et al., 2012). We also include acquisition time, that is, the time elapsed from start of scanning, as movie and traditional localizer tasks occurred at different time points during the scan session (Pixar movie: ~35 minutes into the session versus traditional localizer task run 1: ~17 minutes into the session). Using the mean framewise displacement data from all four runs of the traditional localizer, we conducted a mixed-effects linear regression analysis to model motion including acquisition time and autism diagnosis, with a time-by-group interaction as a fixed effect. We included full-scale IQ, age, and sex as covariates. Because the mean framewise displacement data were right skewed, we applied a Box-Cox transformation to normalize the distribution, applying this transformation to the traditional localizer task data and the Pixar movie data separately. From the resulting regression model of motion from the traditional localizer data, we predicted the amount of motion expected during the Pixar movie. We used a paired one-tailed t-test to compare the actual and predicted motion during the Pixar movie within participants given the hypothesis that the actual motion during the Pixar movie would be less than the predicted motion based on the traditional localizer task data.

We also expected that participants with autism would show higher levels of motion compared to NT participants throughout the scan, but that reductions in motion during the Pixar movie may result in movement levels more consistent with the NT group. To compare motion between groups during the Pixar movie, we fit an ordinary least squares linear regression model to predict Box-Cox-transformed mean framewise displacement between ASD and NT groups, with age, sex, and full-scale IQ included as covariates. We repeated this analysis during each run of the traditional functional localizer task, to test for a group difference.

### 2.6 fMRI data analysis

After analyzing for differences in motion, we then attempted to compensate for motion by removing high motion artifacts. Motion artifacts were defined as timepoints with framewise displacement >0.5. These time points were removed from further analyses; runs with greater than 25% of data lost due to motion were dropped, resulting in 12 dropped runs across the dataset and removal of one NT participant from analyses. Notably, none of the dropped runs were the first run of the traditional localizer task nor the Pixar movie.

Preprocessed data were input into a standard first-level general linear model using the Nilearn package (Abraham et al., 2014) with hrf model set to spm + derivative, high-pass filter of 0.01 Hz, and smoothing set to a full width half maximum of 7.2 mm (3 voxel-width) to produce face-selectivity maps. Head motion was further accounted for by including 24 nuisance regressors derived from rigid-body alignment obtained from fMRIPrep. For the traditional localizer task, we selected the contrast of upright face versus upright house. For the Pixar movie, a previously identified time series (Kamps et al., 2022) was used to contrast movie timepoints which evoke a response to faces in known face-related brain areas compared to ‘other’ movie events. This time series of face events was created using a reverse correlation procedure to identify movie events that produced a reliable BOLD response in face-related brain areas in adults and in children up to age 12. They did so by extracting responses from a pre-defined face area ROI and identifying significant time points, then classifying ‘events’ as two or more consecutive time points. We confirmed that these face events corresponded to spikes in face selective ROIs in our sample, finding a similar time course.

Of note, the analyses reported in this manuscript should be considered exploratory, not confirmatory, because the analyses described here were not chosen prior to data collection, and data collection was not completed with this specific set of analyses in mind. This dataset is associated with K23 MH120510-01, a study assessing face-processing networks in adolescents with and without autism.

### 2.7 Searchlight definition

An *a priori* searchlight region was defined from the Neurosynth ‘face’ meta-analytic result (https://neurosynth.org/), thresholded at z = 5, and trimmed to the temporal lobe. Participant statistical maps of face selectivity were masked with this searchlight, and analyses of peak activation location and pattern reported here are constrained to this masked region. Analyses were conducted bilaterally within participants, to compare the individual functional localization of the FFA from the Pixar movie versus the traditional localizer task in each hemisphere.

### 2.8 Peak activation locations

We first assessed whether the location of the peak voxel, the voxel with the strongest face selective activation, differed between the traditional localizer task and the Pixar movie. We obtained the peak voxel in each participant from the searchlight-masked face selectivity t-maps from the Pixar movie data and from the first run of the traditional localizer task, identifying one voxel in each hemisphere. For consistency, we selected the first run of the traditional localizer task, as every participant completed this run. As a comparison, we determined a “best estimate” FFA peak voxel location by using *all* remaining traditional localizer task data for each participant. To do so, we left out the first run of the traditional localizer task, then combined the face selectivity maps from the remaining three runs of the traditional localizer task in a second-level general linear model using NiLearn (Abraham et al., 2014) with smoothing set to full width half maximum of 2 mm for intra-individual alignment. Due to high motion or inability to collect four task runs from every participant, only 41 participants were included in this analysis (19 ASD, 22 NT). From the resulting face specificity maps, we identified the “best estimate” peak voxel separately within the left and right hemisphere searchlight areas for each participant.

Then, we computed the Euclidean distance from the “best estimate” voxel to the peak voxel derived from the Pixar movie and to the peak voxel derived from the first run of the traditional localizer task within each participant using a paired t-test. Data were approximately normally distributed with no outliers.

Next, we explored whether there were any group differences in FFA peak voxel locations derived from traditional localizer tasks versus the Pixar movie. We conducted a repeated-measures linear mixed-effects model with a within-participants effect of localizer type and autism diagnosis as a between-participants factor. The model included age and sex as covariates to mitigate the effects of differences between groups, since autism is more prevalent in males, as reflected in our sample. We also added full-scale IQ as a covariate and a random intercept for participant. These data met the assumptions for normality and sphericity.

### 2.9 Activation patterns

In addition to comparing the locations of the peak activated voxels from each localizer type, which is a comparison of a single voxel in isolation, we also examined the similarity of overall activation patterns evoked by the traditional localizer task and the Pixar movie. To do so, we calculated the voxelwise pairwise difference between the face selectivity t-map from the first run of the traditional localizer and the Pixar movie face selectivity t-map within participants. Using this difference map, we conducted a permutation based one-sample t-test using PALM (Winkler et al., 2014), that is, a pairwise two-sample t-test, to identify if there were any voxels with a statistically significant activation difference between the traditional localizer task and the Pixar movie, within the searchlight ROI, and across the whole brain. This allows us to both examine overall activation pattern differences and differences in magnitude of activation between the traditional localizer task and the Pixar movie in a pairwise fashion. We also explored if the activation pattern differences between the traditional localizer task and Pixar movie differed between participants with and without autism by conducting a two-sample permutation based t-test using PALM of the face selectivity difference maps between groups. We included mean-centered age, mean-centered full-scale IQ, and sex as covariates.

### 2.10 Application of generated iROIs

To assess whether the FFA localizations were equivalent, we applied resulting individualized ROIs (iROIs; sometimes referred to as subject-specific ROIs/ssROIs; Kamps et al., 2022) from the traditional localizer task and the Pixar movie to an independent run of task data. We created FFA iROIs following the group-constrained subject-specific (GSS) approach (Fedorenko et al., 2010; Julian et al., 2012). Voxels within the Neurosynth searchlight region (described above) were ranked by t-value of the contrast of either face > house (traditional localizer task) or face > other (Pixar movie). The top 200 voxels in each hemisphere were defined as the iROI. As a comparison, we defined a group ROI by drawing a 7 mm sphere around the peak coordinates generated by the Neurosynth result for ‘face’.

To compare the face selectivity resulting from the iROI produced by each localizer type, we compared the responses to faces using data from a single independent run of the traditional face localizer task. For each ROI, we extracted the mean t-score for the contrast of face > house from a GLM analysis using NiLearn (Abraham et al., 2014). We conducted a linear mixed-effects model to compare effects of autism diagnosis and localizer type on mean t-score, with an interaction term and included a random effect of each participant and a covariates of age, full-scale IQ, and sex. These data met assumptions for linearity, normality, and independence of residuals.

## 3 Results

### 3.1 Motion during a Pixar movie was significantly less than predicted by time-in-scanner

**Fig. 1.**
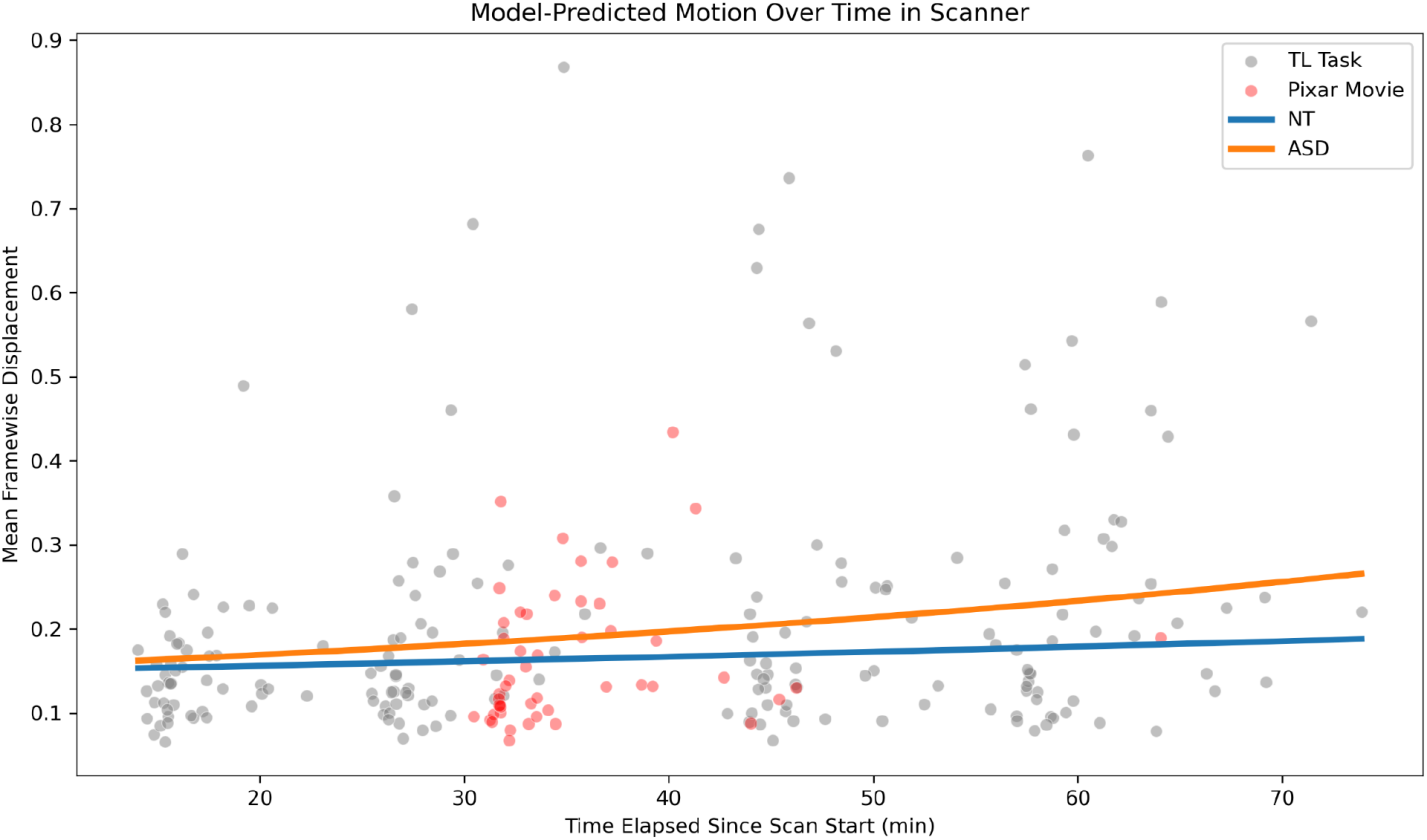
Dots represent the observed motion (mean framewise displacement) during the traditional localizer task (grey) and Pixar movie (red) as a function of time since scan start; lines represent the predicted motion levels using a model fit to traditional localizer task data, plotted against the same time axis. TL: Traditional Localizer.

There was a significant effect of time-in-scanner on motion during task-based fMRI runs, that is, the traditional localizer tasks (β = 0.011, SE = 0.005, z = 2.491, p = 0.013), with motion increasing over the duration of the ~70-minute scan. There was also a slight, but significant, group by time interaction (β = 0.013, SE = 0.006, z = 1.990, p = 0.047) such that autistic participants increased in motion throughout the scan at a steeper rate compared to neurotypical participants (Fig. 1). No significant main effects were observed for age, autism diagnosis, sex, or full-scale IQ **(**all ps > 0.1**)**. Using this model, we calculated the predicted motion during the Pixar movie for each participant based on the length of time since scan start. We found a significant difference, where the actual motion during the Pixar movie was less than predicted (t(48) = -1.76, p = 0.042).

**Fig. 2.**
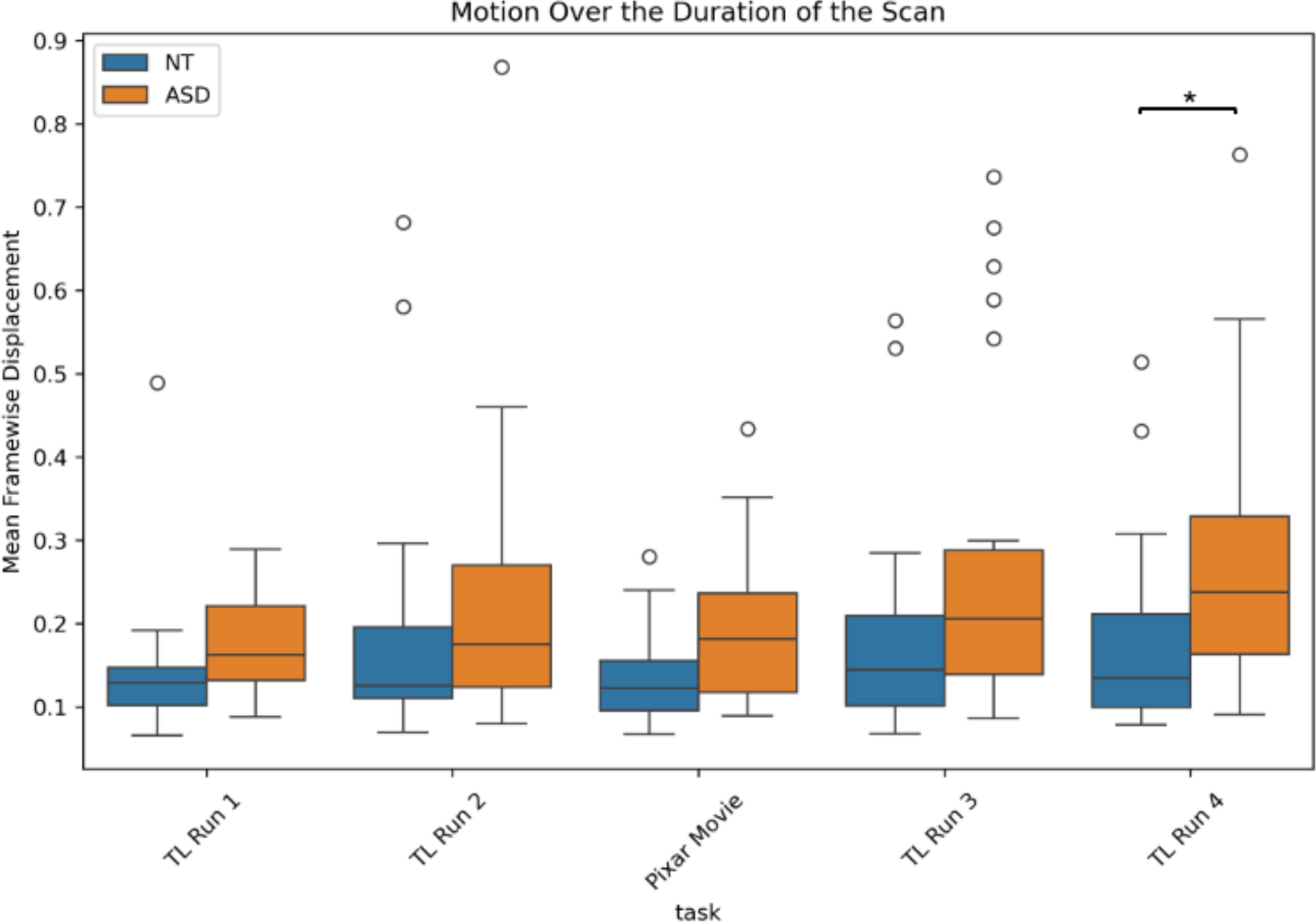
Levels of motion (quantified by mean framewise displacement) across tasks, separated into ASD and NT groups. Motion was significantly greater in the ASD group for TL Run 4 (*p < 0.05).

During the Pixar movie, the ASD and NT groups showed comparable levels of motion (β = 0.08, SE = 0.44, t = 1.81, p = 0.08) (Fig. 2). During the traditional localizer task runs, the ASD group demonstrated significantly higher levels of motion only during the fourth run (β = 1.25, SE = 0.52, t = 2.39, p = 0.02), but no significant differences in motion during the first (β = 0.39, SE = 0.39, t = 1.01, p = 0.32), second (β = 0.12, SE = 0.50, t = 0.25, p = 0.81), or third (β = 0.66, SE = 0.54, t = 1.23, p = 0.23) runs. However, during the second traditional localizer task run, higher IQ was associated with lower motion (β = -0.03, SE = 0.02, t = -2.11, p = 0.04), while sex differences in motion approached significance during the third traditional localizer task run, where male participants moved more than female participants (β = -1.01, SE = 0.51, t = -2.00, p = 0.05).

**Fig. 3.**
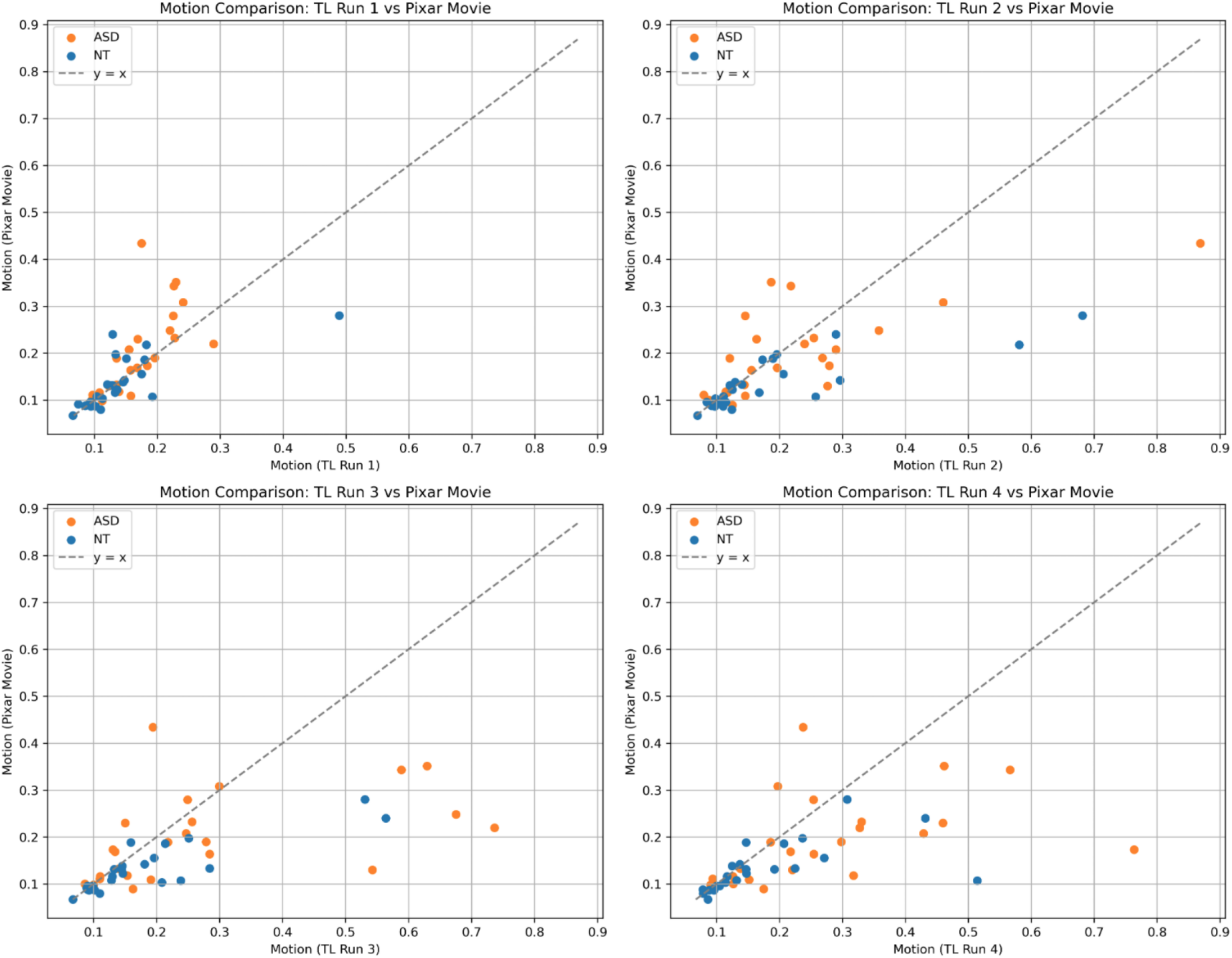
Plots of motion (mean framewise displacement) during the Pixar movie (y-axis) by motion during the traditional localizer (TL) task (x-axis), plotted by individual participant. Points below the line represent participants who moved less during the Pixar movie than during the compared run of the task

To visualize how motion levels differed between traditional localizer task runs and the Pixar movie in individual participants, we plotted motion (mean framewise displacement) for the Pixar movie on the y-axis by motion during a single run of the traditional localizer task on the x-axis (Fig. 3), similarly to a plot from Vanderwal et al. (2019). Points below the line represent participants who moved less during the Pixar movie compared to the traditional localizer tasks; more of these participants appear to be in the ASD group. The Pixar movie does not appear to mitigate motion levels compared to the first run of task, which occurred earlier in the scan session than the Pixar movie, but does appear to help individuals reduce motion compared to task runs 2-4, particularly high movers.

### 3.2 The peak face-selective voxel derived from a Pixar movie was similarly located to the peak face-selective voxel derived from a traditional localizer task

A paired t-test comparing the location of the peak voxel from the traditional localizer task and from the Pixar movie revealed no significant difference (left: t(40) = 0.32, p = 0.75, right: t(40) = 0.44, p = 0.67). A linear mixed-effects model conducted to examine if autism diagnosis influenced peak voxel location across localizer types, while controlling for age, sex, and full-scale IQ, also found no significant main effect of autism diagnosis or localizer type, nor any significant interaction effect between autism diagnosis and localizer type in either the right or left hemispheres (all ps > 0.1). Among the covariates, only full-scale IQ was a significant predictor of the distance between peak voxels, in the left hemisphere (β = 0.286, SE = 0.141, z = 2.026, p = 0.043; right: β = 0.167, SE = 0.10, z = 1.67, p = 0.096).

### 3.3 The Pixar movie and traditional localizer task had a similar activation pattern

**Fig. 4.**
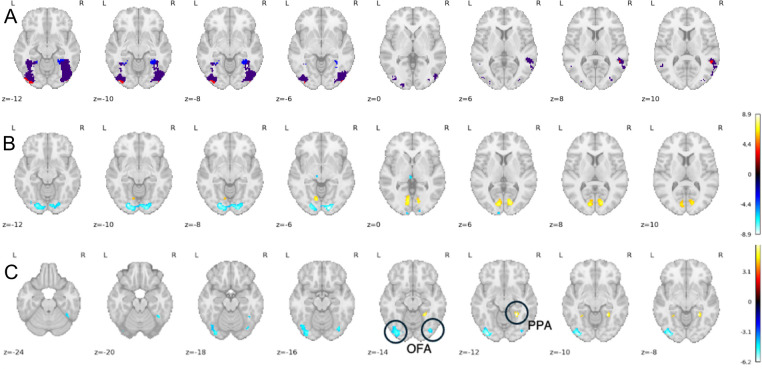
Overlay map (A) showing activation for the Pixar movie (blue), Run 1 of the traditional localizer task (red) and co-activated regions (purple). See Supplementary Figure S1 for non-overlaid activation maps. Whole brain (B) and FFA-masked (C) one-sample permutation analysis of the statistical difference maps for face selectivity (computed independently in each hemisphere then combined for visualization), FWE, p < 0.05. Negative (cool color) regions represent traditional localizer task > Pixar movie. Positive (warm color) regions represent traditional localizer task < Pixar movie. OFA=occipital face area; PPA=Parahippocampal place area. Color bar shows z-statistic.

In a two-sample permutation analysis of the statistical difference maps for face selectivity between clinical groups with age**,** sex, and full-scale IQ included as covariates, no significant voxels survived correction (FWE, p < 0.05). Across the entire group, a one-sample permutation analysis of the statistical difference map for face selectivity revealed 2 significant clusters of voxels (FWE, p < 0.05) in each hemisphere (Fig. 4). The more anterior clusters were activated more during the Pixar movie compared to the traditional localizer task, located nearby the parahippocampal place area (PPA). The more posterior clusters were activated more during the traditional localizer task than the Pixar movie, located nearby the occipital face area (OFA).

A whole-brain one-sample permutation analysis of the statistical difference map for face selectivity revealed regions in primary visual cortex and other traditionally facial/visually responsive areas that were activated significantly more during the Pixar movie compared to the traditional localizer task and vice versa (FWE, p < 0.05; Fig. 4).

### 3.4 The traditional localizer produced the most face selective responses

**Fig. 5.**
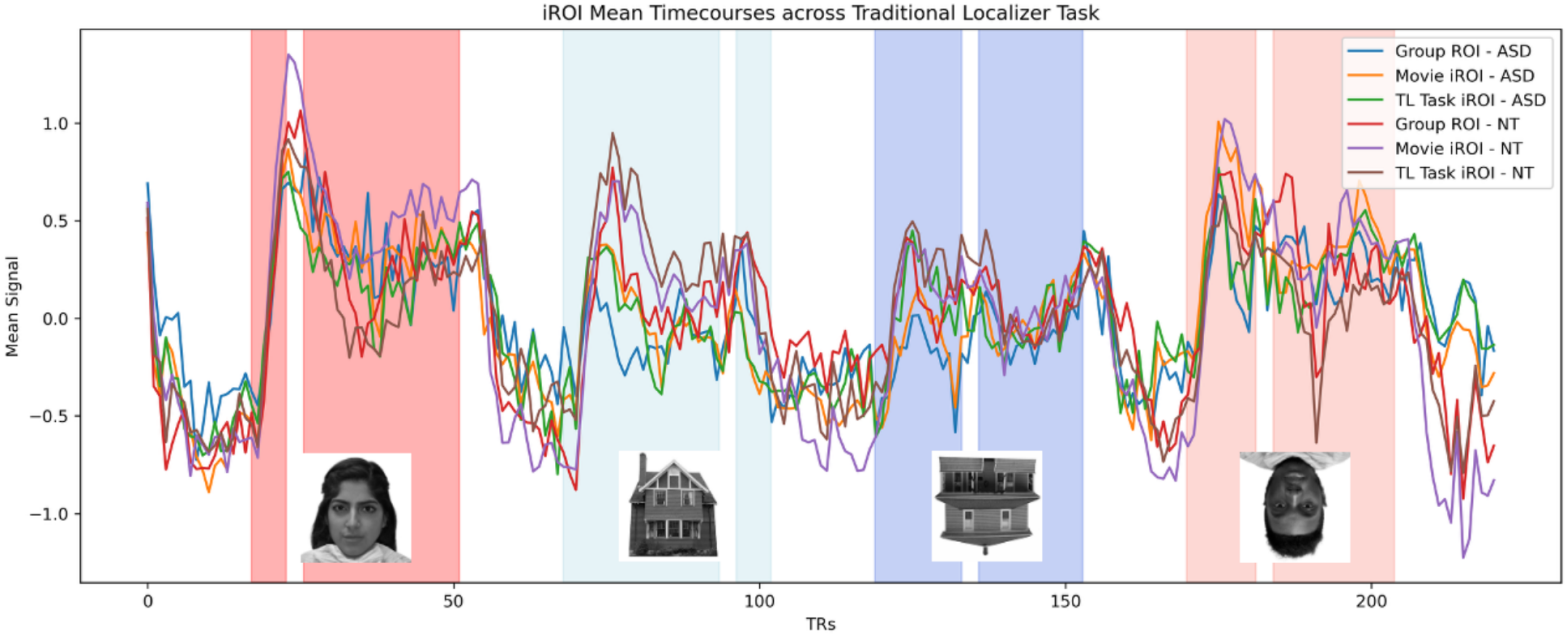
Z-normalized mean timecourses from each ROI type applied to an independent run of the traditional localizer task in the left hemisphere, across ASD and NT groups. Right hemisphere time course looks very similar and so is not pictured here. White columns within blocks indicate presentation of flower stimulus.

The Pixar movie iROIs produced an appropriately appearing time course, with face selective spikes, as did the traditional localizer task iROI and Neurosynth ROIs (Fig. 5).

**Fig. 6.**
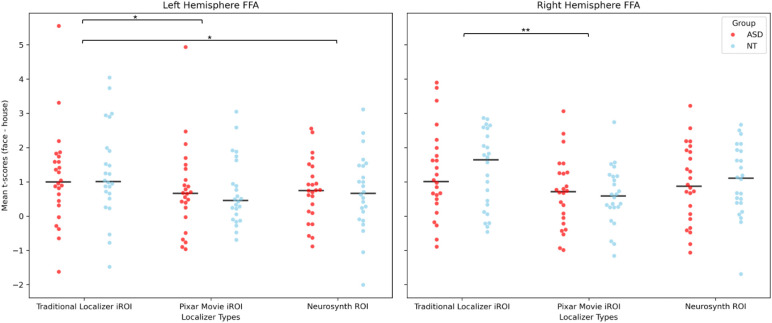
Distribution of mean t-scores for face selectivity from each ROI type, applied to an independent run of the traditional localizer faces versus houses task, separated by group. Traditional Localizer task iROIs outperformed Pixar Movie iROIs in both hemispheres, and outperformed Neurosynth ROIs in the left hemisphere (*p < 0.05, **p < 0.01). No group differences.

A linear mixed-effects model comparing autism diagnosis and ROI types revealed no significant interactions and no significant main effects of age, sex, or full-scale IQ (all ps>0.4), but a significant main effect of ROI type on mean face selective activation. The iROI from the traditional localizer resulted in significantly greater face selective activation than the iROI from the Pixar movie (left: β = -0.363, SE = 0.175, z = -2.077, p = 0.038; right: β = -0.542, SE = 0.180, z = -3.018, p = 0.003) and the group ROI from Neurosynth in the left hemisphere only (left: β = -0.377, SE = 0.175, z = -2.157, p = 0.03; right: β = -0.284, SE = 0.18, z = -1.59, p = 0.11) (Fig. 6). The Pixar movie iROI was not significantly different from the Neurosynth ROI in either hemisphere (left: β = -0.01, SE = 0.18, z = -0.08, p = 0.94; right: β = 0.26, SE = 0.18, z = 1.43, p = 0.15). There were no main effects of group (left: β = 0.02, SE = 0.41, z = 0.06, p = 0.96; right: β = -0.02, SE = 0.37, z = -0.05, p = 0.96).

## 4 Discussion

Here, we have demonstrated that highly engaging naturalistic stimuli, like a Pixar movie, can be a suitable choice to replace traditional functional localizers for stimuli-selective regions such as the FFA, with particular benefit for developmental clinical populations. Our analysis confirms and extends previous work suggesting that movies, such as *Partly Cloudy*, can be used effectively as a functional localizer in adults and in young children (Kamps et al., 2022). Our results find that, in adolescents with and without autism, a Pixar movie localizes face selective regions largely consistent with those produced by the traditional localizer, indicating that it is a valid choice for an FFA functional localizer. When applied to an independent run of the traditional localizer task, the task-derived iROI produced a stronger activation than the movie-derived iROI, suggesting that a traditional localizer task is still better at identifying the most face selective brain regions. We also demonstrated that the Pixar movie helps to reduce motion levels across our sample relative to task localizers. While the FFA was still localized in all individuals during the task and movie, this decrease in motion may offer an opportunity to both shorten scan time and increase scan engagement, further increasing scan accessibility and tolerability for populations at risk for extreme motion or opting out of fMRI scans altogether. Our results point to further investigation of movie use as functional localizers in other clinical and/or developmental populations.

We found that the FFA peak activation locations were consistent between the Pixar movie and the traditional localizer task, in both hemispheres. We also found that the FFA activations were similar in both pattern and magnitude, with the exception of two identified clusters where the Pixar movie and traditional localizer task differed in each hemisphere. A more posterior bilateral cluster, appearing to represent the occipital face area (OFA), was significantly more activated by the traditional localizer task than by the Pixar movie. In clinical studies of prosopagnosia and TMS stimulation experiments, the OFA has been demonstrated to be important alongside the FFA for face perception, where disruption resulted in impaired judgement of gender and emotion (Müller et al., 2018). Other work suggests that the OFA is situated in an early position in a hierarchical model of face processing and is specifically crucial for the extraction and processing of local features, such as eyes, ears, and nose (Pitcher, Walsh, et al., 2011). The features of the Pixar movie—expressive, animated, anthropomorphic faces involved in a narrative—that make it entertaining and enable simultaneous localization of different functional regions may also contribute to this difference in OFA activation, compared to the traditional localizer task consisting of novel, neutral human faces. Previous research using this Pixar film did not investigate the OFA, because a reliable face selective response could not be identified in a large sample of adults used to create group level searchlight ROIs for each functional region (Kamps et al., 2022).

A more anterior cluster was significantly more activated by the Pixar movie compared to the traditional localizer task; this appears to represent the parahippocampal place region (PPA), which activates preferentially to place stimuli (Epstein & Kanwisher, 1998). This difference was likely due to the fact that faces are embedded in spatial scenes in the Pixar movie stimuli compared to the isolated face stimuli in the traditional faces versus houses task. This highlights both a challenge and an advantage of using naturalistic tasks; overlapping stimuli presentations can result in complex timecourses and simple contrast modeling may have difficulty in parsing them apart, yet this inherently provides a way to simultaneously localize multiple regions from one “task”. For example, this same Pixar movie was used to localize pain and theory of mind networks, as well as place, object, and face regions in both young children and adults (Richardson et al., 2018). This reinforces that using a single run to localize multiple functional regions or networks in individuals is an effective way to maximize expensive scan time and make scanning more efficient for populations which may not tolerate lengthy paradigms.

While visual inspection of the time courses from each iROI indicates consistent face selective activation (Fig. 5), there were some differences in face-selective activation between the traditional localizer iROIs, Pixar movie iROIs, and the Neurosynth ROI. Similar to results from Kamps et al. (2022) in adults and young children, the traditional localizer outperformed the Pixar movie in producing the most specific face selective iROIs. However, while they found that the Pixar movie iROIs outperformed the group ROI, we did not find a significant difference in face selective activation. This may be due to differences in the size of the group-level ROIs; while we used ROIs consisting of a 7 mm sphere surrounding the peak voxel coordinate of the Neurosynth ‘face’ result, Kamps et al. (2022) used a much larger group-level ROI from a large independent sample of adults. The larger ROI increases the comparison voxels and may have decreased the specificity of the signal in their work, thereby making it less selective than the Pixar movie iROI. Given these results, in a situation where functional localization from a traditional task localizer is a limiting factor, it may be useful to use an ROI generated from a large, meta-analytic fMRI database, like Neurosynth, for a group-level ROI approach. It may also be possible that our results differ because Kamps et al. (2022) conducted their comparison of group-, task-, and movie-derived iROIs in a small sample of adults, then correlated time courses from movie iROIs in a large sample of children to the time courses from task-derived iROIs in adults. Conducting our iROI comparison directly in a developmental, clinical population may contribute to lower data quality than in an adult sample, which would also make it more difficult to detect differences. Our somewhat differing results call for future research investigating differences in functional localizations derived from independent groups, tasks, and movies directly in larger clinical and developmental populations to extend from our conclusion that movies are *sufficient*, to determine what is the *optimal* localizer in different cohorts.

We found no differences between ASD and NT participants in FFA localization or face selectivity. Given the mixed findings in the literature regarding FFA neural responses in autism and the small size of our study, this result is not surprising and should not be taken as evidence of “no difference”. Hypoactivation of the FFA in autism has been sometimes associated with differences in task demands, where passive viewing tasks result in comparable activation between NT and ASD (Hadjikhani et al., 2004, 2007; Okamoto et al., 2017), but active tasks result in FFA hypoactivation in ASD (Critchley et al., 2000; Humphreys et al., 2008; Kleinhans et al., 2010; Pierce, 2001; Piggot et al., 2004; Pinkham et al., 2008; Schultz et al., 2000). Meanwhile, others have posited that autism-related FFA hypoactivation is related to autism symptom level, suggesting that studies reporting similar FFA activation between NT and ASD included a large percentage of participants with a DSM-IV Asperger’s Syndrome diagnosis, now often termed “high-functioning” ASD, while studies reporting hypoactivation in ASD included only patients with a DSM-IV ASD diagnosis (Scherf et al., 2015). Of note, Scherf and colleagues (Scherf, 2010; Scherf et al., 2015) demonstrated that adolescents with more severe autism symptoms as measured by the Social Responsiveness Scale exhibited less face-related FFA activation. Our sample of autistic adolescents was likely self-selected for participants with lower support needs (“high-functioning”) due to the study requirement of a 1-hour fMRI scan, and so it would be consistent with the literature that we saw no significant differences in face-related FFA activation. Nonetheless, similar overall metrics with a faster, less-arduous protocol makes movie-based localizers a practicable option for studies of ASD regardless of symptom severity, particularly if able to be tailored to the participants’ interests.

Another motivation in using a movie as a functional localizer over a traditional localizer task is to increase engagement and reduce high motion when studying groups which struggle to participate in lengthy scan protocols. Here, we found that all participants’ motion levels were lower during movie watching than predicted by our model assuming sequential task data. This finding differs from previous research in large cohorts showing that mean framewise displacement does not typically improve during movie watching relative to tasks in adolescents, even though it had been shown to be helpful for younger children (Frew et al., 2022; Greene et al., 2018; Vanderwal et al., 2019). While encouraging, our sample is relatively small and this finding would need to be replicated in larger cohorts. On an individual level, “high movers” during traditional localizer tasks appeared to show a larger reduction in motion during movie watching, while “low movers” during tasks demonstrated less change in motion during movie watching (Fig. 3). This is consistent with prior work investigating motion in a large developmental sample (Frew et al., 2022) and encourages that movies may be a good strategy to consistently collect higher quality data from high-moving participants. High movers, who were mostly individuals with ASD, appeared to benefit more from movie watching than low movers, who tended to be NT peers, which is consistent with prior work demonstrating differences in motion levels between autistic and neurotypical individuals (Dosenbach et al., 2017; Martin et al., 2018; Nebel et al., 2022; Yerys et al., 2009).

Overall, we did not find group differences in head motion during most task runs, finding increased motion in autistic individuals only during the last traditional localizer run. This motion difference at the end of a long fMRI scan session likely drives the overall steeper head motion increase for the ASD group over the duration of the scan. Thus, efforts to limit scan length or replace sequences with engaging movie viewing are particularly beneficial to reduce confounding motion-related differences between groups which emerge during longer scan sessions. While there were only group-related motion differences in the last traditional localizer run, IQ- and sex-related differences for motion emerged during middle task runs (Run 2 and Run 3, respectively), but not during the Pixar movie, also occurring in the middle of the scan. Previous work investigating motion in a large-scale developmental sample found higher rates of motion in males than females (Frew et al., 2022). Autism is more prevalent in males, so perhaps autism-related motion increases are at least in part accounted for as sex-related motion increases. Our findings appear to complement conclusions of prior work investigating motion in large developmental populations (Dosenbach et al., 2017; Frew et al., 2022) in that head motion is driven by individual factors, and likely it is a combination of psychopathology and behavioral factors related to autism, as well as demographic (i.e., sex, age) or cognitive factors (i.e., IQ) that underlie trait-level differences in motion levels. Overall, this suggests that movie watching is a helpful motion mitigator *in general*, not only in relation to increases in motion observed in autistic populations.

While our findings support the use of engaging movie watching as a functional localizer across the developmental age range, there are a few notable limitations to this study. First, in our analysis comparing the peak activated voxel locations, the “best estimate” peak voxel was derived from the same task as the traditional localizer task, albeit from more than twice the amount of data and from separate BOLD runs. In an ideal set-up, this “best estimate” of the FFA would be computed from an independent task. While “circular”, this comparison only biases *against* the Pixar movie, so the relative equivalency of results from movie watching despite this bias is encouraging. Second, because these are exploratory analyses, this dataset was collected without this specific analysis in mind and so the traditional localizer task and Pixar movie are not counterbalanced in our task design. The Pixar movie is played once in the middle of the scan session for all participants. Ideally, the order of task and Pixar movie would be counterbalanced across participants, to control for effects related to task order, and the number of movie runs to task runs would be matched, to control for the novelty of the Pixar movie. We attempt to account for this in our motion analyses by including a time variable, but this does not fully capture the novelty of the Pixar movie in comparison to the traditional localizer task. Additionally, our sample size is comparatively small by modern standards and may be underpowered for diagnosis-level interpretations; conclusions regarding autism versus neurotypical groups beyond our motion analysis should be interpreted with caution.

Finally, when using naturalistic stimuli, there is not a standard convention for defining ‘events’ like there is for more traditional fMRI tasks. We elected to use timing for face events from previous work using this same Pixar movie. As this is not always possible, researchers can use alternative approaches, such as a data-driven reverse correlation method or a machine-learning model to identify events of interest. We explored various machine learning models to identify face events directly from the Pixar movie, finding that pre-trained models were trained on human face data and did not generalize well to cartoon anthropomorphic animal and cloud faces. The best approach would be to fine-tune or train a new deep learning model on task-specific data. Given the lengthy nature of this approach, we tried a model based on zero-shot object detection (Liu et al., 2024). Considering that the model uses raw unlabeled data, we were surprised to find that the face events were similar to those that we used, identified by Kamps et al. (2022) (Supplementary Fig. S2). Ultimately, we opted to use the face events identified in prior work so that we could make a more direct comparison. Yet, this poses a question for future work to compare methods for defining events from naturalistic data, including various available pre-trained, fine-tuned, one-shot, or zero-shot models.

In summary, using an engaging and entertaining short movie, such as *Partly Cloudy*, for functional localization appears to be an appropriate choice in populations which may be undersampled in fMRI research due to greater levels of scan fatigue in the stressful MRI environment, such as individuals with autism, or in any paradigm in which multiple functional regions need to be rapidly localized in individuals.

## Supplementary Material

Supplementary Material

## Data Availability

Data and code are available upon request.
