# Supplementary material for: Naturalistic movie viewing is an effective functional localizer of the fusiform face area in adolescents with and without autism
Source: Imaging Neurosci (Camb). 2026 Apr 8;4:IMAG.a.1209. doi: 10.1162/IMAG.a.1209 (PMC13062985; doi:10.1162/IMAG.a.1209)
Supplement: Supplementary Material [file IMAG.a.1209_supp.pdf]

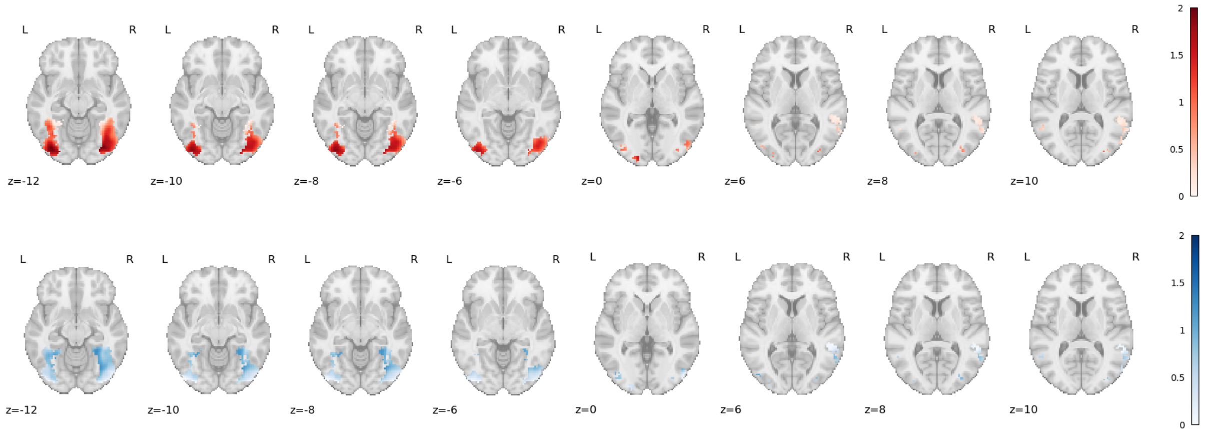

Supplemental Figure 1: Blue regions represent areas significantly activated during the Pixar movie. Red regions represent areas significantly activated during Run 1 of the traditional localizer task. Color bar shows z-statistic.

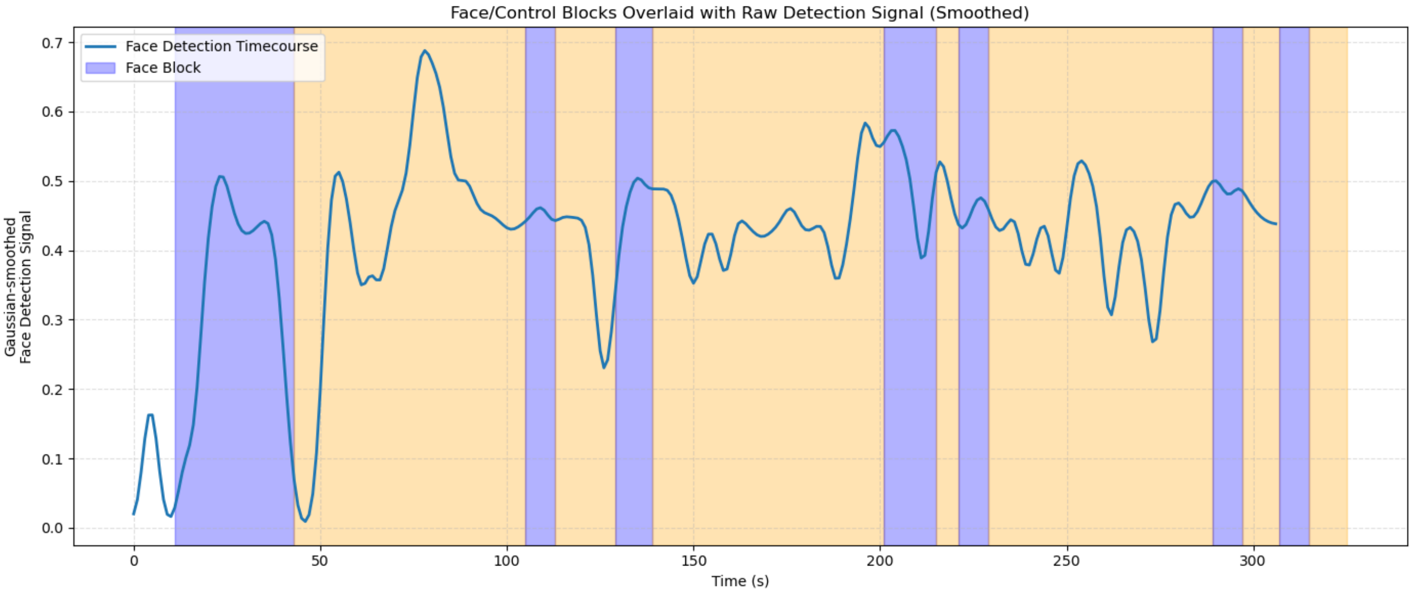

Supplemental Figure 2: Line shows face detection during the Pixar movie by a zero-shot model (cite) compared to face events as defined in Kamps et al. (2023) and used in the current experiment. Model face detection is Gaussian-smoothed at sigma=2.
